# Supplementary material for: Functional reorganisation of the cranial skeleton during the cynodont–mammaliaform transition
Source: Commun Biol. 2023 Apr 12;6:367. doi: 10.1038/s42003-023-04742-0 (PMC10097706; doi:10.1038/s42003-023-04742-0)
Supplement: Supplementary file 2 — Supplementary Material [file 42003_2023_4742_MOESM2_ESM.pdf]

Supplementary Information for:

Functional reorganisation of the cranial skeleton during the cynodont-mammaliaform transition

Stephan Lautenschlager, Michael J. Fagan, Zhe-Xi, Luo, Charlotte M. Bird, Pamela Gill[4], Emily J. Rayfield

Supplementary Note 1: Digital restoration and reconstruction of specimens

Supplementary table 1. Measurements used in Fig. 1

Supplementary table 2. Calculated Euclidean distances used in Fig. 5

Supplementary figure 1. Combined stress values

Supplementary figure 2. Sensitivity results for *Hadrocodium*

Supplementary figure 3. Landmark positions

Supplementary Note 1: Digital restoration and reconstruction of specimens

This section provides a description of the digital restoration and reconstruction steps performed for the specimens used in this study and as figured in the main text. Due to the often incomplete preservation of the physical specimens, digital restoration steps were required to remove taphonomic artefacts before the jaw adductor musculature was digitally reconstructed and further biomechanical analyses were performed. For the restoration process of the different models the scan data were imported into Avizo (VSG, Visualisation Science Group, France). Data derived from CT scanning were segmented manually to isolate fossilised bone from surrounding matrix utilising the Avizo segmentation editor. To remove taphonomic artefacts and to restore the original in-vivo condition as closely as possible, different digital restoration steps were applied (Lautenschlager, 2016).

Cracks and small breaks were removed manually during the segmentation process by interpolating across the affected region. Unilaterally missing regions and elements were restored by reflecting preserved counterparts across the bilaterally symmetrical long axis of the skull. Rearticulation was performed in systematic order with the least deformed bones first using articulation facets and the general skull topology as a guide. Missing elements were supplemented by using information of other specimens or closely related species. Where necessary, plastic deformation was repaired by employing a landmark-based retrodeformation approach performed with the geometric morphometrics software Landmark (version 1.6, www.idav.ucdavis.edu/research/EvoMorph). For that purpose, corresponding, bilaterally symmetric landmarks were selected on both sides of the specimen. Based on the distance between landmarks the plane of symmetry was calculated by the software. This information was subsequently used to warp and symmetrise the model. Further, model-specific restoration steps and scanning details are listed below.

Thrinaxodon liorhinus

For the model of *Thrinaxodon liorhinus*, CT scans of the holotype skull (NHMUK PV R 511, Natural History Museum, London, UK) and mandibles (NHMUK UK PV R 511a) were used. Scans were performed with a Nikon Metrology HMX ST 225 CT scanner set at 190 kV and 135µA for the skull and 180 kV and 150µA for the mandibles. The final image stacks (1923×1348×804 pixels, 50.2 µm voxel size for the skull; 1876×1661×433 pixels, 44.3 µm voxel size for the mandibles) were imported into Avizo (version 8, VSG) and segmented manually using a combination of the paintbrush tool, magic wand tool, and interpolation. The digital model of the skull and lower jaw required only minor restoration. The specimens are well preserved and nearly complete. Breaks and small fractures were removed during the segmentation process. As the left jugal is only fragmentarily preserved, the complete right element was used to produce an antimere. Both canine teeth in the skull are missing in NHMUK PV R 511 and were supplemented by from a similar-sized, second specimen (BSP 1934 VIII 506, Bayerische Staatssammlung für Historische Geologie und Paläontologie, Munich) of *Thrinaxodon liorhinus*.

Diademodon tetragonus

The specimen used in this study (BSP 1934 VIII 17/2) was originally described as *Diademodon* (*Gomphognathus*) *grossarthi* (Broili & Schröder, 1935; Brink, 1979), now regarded as an invalid synonym of *Diademodon tetragonus* (Martinelli *et al.,* 2009). The skull and lower jaw of BSP 1934 VIII 17/2 were CT scanned at the Zoologische Staatssammlung Munich using a Nanotom m (phoenix|x-ray) CT scanner set at 180 kV and 120µA. The final image stacks (1500×1500×1200 pixels, 78.2 µm voxel size) were imported into Avizo (version 8, VSG) and segmented manually using a combination of the paintbrush tool, magic wand tool, and interpolation. Although the good state of preservation and size of BSP 1934 VIII 17/2 permitted the creation of a detailed

and accurate digital model, some minor taphonomic artefacts required correction. Breaks and cracks were removed during segmentation. The tooth crowns of both canine teeth in the skull were not preserved in the specimen and were reconstructed using the mandibular canine teeth and the size and position of the mandible and mandibular dentition as guidance.

Chiniquodon sanjuanensis

The specimen of *Chiniquodon sanjuanensis* PVSJ 411 (originally assigned to the genus *Probelesodon)* used in this study consists of a nearly complete articulated skull and a mostly complete, attached mandible. CT scans of the specimen were provided by Dr T. Rowe as part of the Digimorph project (http://digimorph.org/specimens/Probelesodon_sanjuanensis/). The specimen was originally scanned at the University of Texas High-Resolution X-ray CT Facility, Austin, Texas, USA. The final image stacks (512 × 512 × 402 pixels, 200 µm voxel size) were imported into Avizo (version 8, VSG) and segmented manually using a combination of the paintbrush tool, magic wand tool, and interpolation.

As the specimen is largely complete and undistorted, PVSJ 411 required only little digital restoration. PVSJ 411 had been partially prepared, but a large part of the internal anatomy is still embedded in calcareous matrix, which had to be removed during the segmentation process, but also obscured some fine details in the CT data set. The left squamosal is only partially preserved and was supplemented by the right squamosal. The anterior snout region has been substantially eroded. As a consequence, the premaxillary processes had to be reconstructed on the basis of comparisons with other species of *Chiniquodon* (Abdala & Giannini, 2002).

Probainognathus jenseni

For the digital model of *Probainognathus* CT scans of PVSJ 410 (Museo de Ciencias Naturales, Universidad Nacional de San Juan, Argentina) were made available by Dr T. Rowe as part of the

Digimorph project (http://digimorph.org/specimens/Probainognathus_sp/). The specimen was originally scanned at the University of Texas High-Resolution X-ray CT Facility. The final image stacks (512 × 512 × 496 pixels, 85 µm voxel size) were imported into Avizo (version 8, VSG) and segmented manually using a combination of the paintbrush tool, magic wand tool, and interpolation. The specimen likely represents an immature individual referred to as *Probainognathus* sp. (Bonaparte & Crompton, 1994), which shows more pronounced mammal-like characters compared to adult specimens, including a relatively larger braincase, a slender zygomatic arch, and post-canine teeth morphologically similar to those of *Morganucodon* although different from *Morganucodon* in lacking the precise dental occlusion of the upper and lower post-canines (Kemp, 2005). The fully erupted dentition indicates that the specimens was in an ontogenetic stage that allowed independent feeding, providing a valid model for the biomechanical analyses. While the ontogenetic status of this specimen has precluded a precise taxonomic identification, this has no influence on the reconstructed morphology of the specimen and the functional analyses in this study. However, caution is warranted if evolutionary hypotheses are made focused on this specimen alone.

PVSJ 410 consists of an articulated skull and lower jaw and is largely complete, except parts of the left side, including the jugal, postorbital, squamosal, quadrate and most of the post-dentary region are missing. The cranial restoration was , therefore, primarily based on the complete right side. The right postorbital had been displaced anteriorly and ventrally and had to be rearticulated according to the articulation facets on the jugal and the general dimensions of the postorbital region. Although the specimen had been prepared externally, parts of the braincase and the narial and palatal region were still embedded in matrix. Using the CT data is was possible to remove the matrix digitally. However, due to the preservation and low scan resolution, not all the morphology of the affected regions could be restored, resulting in some gaps in the palate and the

anterolateral braincase wall. The gaps were closed manually by connecting the adjacent bone surfaces.

<u>*Morganucodon oehleri*</u>

The digital model of *Morganucodon oehleri* is largely based on CT scans of an articulated skull and mandible (holotype FMNH CUP 2320, Field Museum of Natural History, Chicago, USA). FMNH CUP 2320 was CT scanned at the University of Chicago using a General Electrics v—tome—x s 240 scanner. Scan parameters were set at 110 kV and 80µA. CT data of a second specimen (IVPP 8685, Institute for Vertebrate Palaeontology and Palaeoanthropology) and additional disarticulated elements of *Morganucodon watsoni* (NHMUK PV M 26144, articulated squamosal and petrosal; NHMUK PV M 92838 & M 92843, isolated quadrates; NHMUK PV M 27410, isolated fragmentary jugal; Natural History Museum, London, UK) were further used for the model creation. IVPP 8685 was scanned at the University of Texas High-Resolution X-ray CT Facility with parameters set at 150 kV and 38µA. All other disarticulated elements were scanned at the University of Bristol using a Bruker SkyScan1272 Micro-CT scanner with parameters set at 166 kV and 60µA. Final image stacks for FMNH CUP 2320 (1600×1600×3576 pixels, 10.7 µm voxel size), IVPP 8685 (1024×1024×960, 25.7 µm voxel size) and NHMUK elements (2452×2452×1106 pixels, 5.1 µm voxel size) were imported into Avizo (version 8, VSG) and segmented manually using a combination of the paintbrush tool, magic wand tool, and interpolation.

FMNH CUP 2320 represents the most complete and best-preserved specimen for any *Morganucodon* species and was used mainly for the restoration process. Although it consists of an articulated and mostly complete skull and isolated right mandible, the specimen has suffered considerably from taphonomic processes and erosion. The left side of the specimen has been eroded more substantially. Therefore the restoration focussed to a large part on the more completely preserved right side. The individual elements were separately segmented using Avizo.

Small breaks and cracks were removed during the segmentation process by interpolating along the complete bone margins. The left premaxilla, the right maxilla, the right lacrimal and the right frontal, post repair, were mirrored across the bilaterally symmetrical long axis of the skull to produce their antimere. The proportions of each element and of the completed skull and mandible models were compared throughout to those of IVPP 8685 and pre-existing reconstructions to ensure consistency. As neither FMNH CUP 2320 nor IVPP 8658 preserve the squamosal, the quadrates and the jugals, these elements were supplemented by isolated specimens of *Morganucodon watsoni* (NHMUK PV M 26144, articulated squamosal and petrosal; NHMUK PV M 92838 & M 92843, isolated quadrates; NHMUK PV M 27410, isolated fragmentary jugal). All elements were rearticulated using Avizo. As the jugal is only partially preserved in any known specimen of *Morganucodon*, parts of the zygomatic arch had to be interpolated between the fragmentary anterior portion of the jugal and the squamosal. This step was performed manually with the preserved elements as a guide. The curvature of the zygomatic arch was modelled so that it created a natural outline without bowing too much laterally or medially. Similarly, the orbitosphenoid region between the frontal, lacrimal and the braincase elements had to be reconstructed, as this region is not preserved fully. The reconstruction was based on the topological constraints provided by the surrounding and articulating bones and published reconstructions (Kermack et al. 1981).

Hadrocodium wui

For the digital model of *Hadrocodium wui,* CT data of a nearly complete skull and articulated lower jaw (IVPP 8275; Institute for Vertebrate Palaeontology and Palaeoanthropology, Beijing) were used. The specimen was scanned at the University of Texas High-Resolution X-ray CT Facility using a XYZ scanner with parameters set at 150 kV and 38µA. The final image stack (1024×1024×735 pixels, 19 µ mvoxel size) was imported into into Avizo (version 8, VSG) and

segmented manually using a combination of the paintbrush tool, magic wand tool, and interpolation. The specimen is comparably complete and consists of an articulated skull, and most of the lower jaws. However, the specimen has suffered considerably from taphonomic deformation, which caused the skull roof around the parietals and frontals to collapse inwards. For the restoration process, the individual elements were segmented separately and breaks and cracks were removed using Avizo. The rearticulation of the individual elements was performed in a systematic order with the elements and regions affected least by taphonomy articulated first. This allowed a rearticulation of the displaced frontal and parietal bones, which were rotated and translated in Avizo to create a consistent morphology of the skull roof. Due to erosion and other taphonomic processes, IVPP 8275 is missing parts of the zygomatic arch and of the narial process of the premaxilla. Similar to the restoration process of *Morganucodon*, the regions were interpolated manually as no other specimen of *Hadrocodium wui* exists. The zygomatic arch was reconstructed between the anterior portion of the jugal and the squamosal with a minimum of curvature to create a natural outline. The same principle was applied for the narial process of the premaxilla. Similar to the condition found in *Morganucodon*, the orbitosphenoid is not preserved and had to be reconstructed in *Hadrocodium wui* on the basis of topological criteria and published reconstructions (Luo et al., 2001). The tip of the coronoid process of the mandible are incompletely preserved, indicated by the uneven, posterior edges on the left and right hemi-mandible. As no other specimen of *Hadrocodium wui* exists, the morphology of the coronoid was reconstructed similar to that of *Morganucodon oehleri* and existing, two-dimensional reconstructions (Luo et al., 2017). Although originally reported to be lacking a post-dentary trough (Luo et al. 2001), new analyses of IVPP 8275 have shown that a small post-dentary trough is actually present in *Hadrocodium wui* (Luo et al., 2016, 2017).

In this context, it should be noted that a large part of the zygomatic arch of *Hadrocodium wui* is not preserved and had to be reconstructed for this study. Due to the lack of further specimens,

which could offer information on its morphology, the zygomatic arch was reconstructed in the most parsimonious way as a straight connection between the incomplete squamosal and jugal.

<u>*Monodelphis domestica*</u>

Monodelphis domestica (Gray short-tailed opossum) was chosen as an extant insectivorous representative and comparative specimen. For this study, a frozen, but otherwise intact specimen of *Monodelphis domestica* was obtained on loan from the National Museum of Scotland, Edinburgh (Z.2013.185.1). The specimen was CT scanned at the μ-VIS facility of the University of Southampton using a Nikon Metrology HMX ST 225 CT scanner with parameters set at 150 kV and 60μA. The final image stack (2000×2000×800 pixels, 33 μm voxel size) was imported into Avizo (version 8, VSG) and segmented manually using a combination of the paintbrush tool, magic wand tool, and interpolation. As an extant specimen no further retrodeformation or reconstruction steps were required.

<u>*Dasyurus hallucatus*</u>

Dasyurus hallucatus (Northern quoll) (TMM M-6921, Texas Memorial Museum) was chosen as an extant carnivorous representative and comparative specimen. The CT data of the specimen was provided by Dr T. Rowe as part of the Digimorph project (available here http://digimorph.org/specimens/Dasyurus_hallucatus). The specimen was originally scanned at the University of Texas High-Resolution X-ray CT Facility, Austin, Texas, USA. The final image stacks (512 × 512 × 780 pixels, 78 μm voxel size) were imported into Avizo (version 8, VSG) and segmented manually using a combination of the paintbrush tool, magic wand tool, and interpolation. As an extant specimen no further retrodeformation or reconstruction steps were required.

Petropseudes dahli

Petropseudes dahli (Rock ring tail possum) (AMNH 183391, American Museum of Natural History) was chosen as an extant herbivorous representative and comparative specimen. The CT data of the specimen was provided by Dr T. Rowe as part of the Digimorph project (http://digimorph.org/specimens/Petropseudes_dahli). The specimen was originally scanned at the University of Texas High-Resolution X-ray CT Facility, Austin, Texas, USA. The final image stacks (1024 × 1024 × 1432 pixels, 48 µm voxel size) were imported into Avizo (version 8, VSG) and segmented manually using a combination of the paintbrush tool, magic wand tool, and interpolation. As an extant specimen no further retrodeformation or reconstruction steps were required.

Taxon	Relative zygoma height	Relative braincase width
Procynosuchus	0.085641026	0.098076923
Galesaurus	0.153846154	0.123589744
Platycraniellus	0.167948718	0.138076923
Thrinaxodon	0.124358974	0.097564103
Cynognathus	0.199615385	0.124615385
Diademodon	0.203333333	0.114102564
Trirachodon	0.184230769	0.112564103
Pascualgnathus	0.205384615	0.117179487
Massetognathus	0.178076923	0.09
Exaeretodon	0.206282051	0.137435897
Exteninion	0.174487179	0.16474359
Chiniquodon	0.232564103	0.167820513
Probainognathus sp.	0.106666667	0.115384615
Oligokyphus	0.159871795	0.105512821
Tritylodon	0.227692308	0.101794872
Kayentatherium	0.222051282	0.145384615
Brasilitherium	0.098076923	0.123846154
Brasilodon	0.053333333	0.155128205
Megazostrodon	0.058205128	0.188974359
Morganucodon	0.069871795	0.228333333
Sinoconodon	0.053333333	0.190128205
Hadrocodium	0.055641026	0.315512821
Gobiconodon	0.101538462	0.12025641
Repenomamus	0.159871795	0.166538462
Jeholodens	0.058974359	-
Rugosodon	0.048461538	0.221282051
Zhangheotherium	0.087179487	0.200641026
Maotherium	0.069358974	0.305
Eomaia	0.038717949	-
Monodelphis	0.075862069	0.25862069
Petropseudes	0.113676732	0.373001776
Dasyurus	0.070546737	0.358024691

Supplementary table 1. Measurements for the relative height of the zygoma (height measured at the dorsoventrally highest point/skull length) and the relative width of the braincase (width measured at the mediolaterally widest point of the braincase/skull length) as used in figure 1 of the main text.

	Region 1	Region 2	Region 3	Region 4	Region 5	Region 6
Thrinaxodon liorhinus						
unilateral canine bite	0.010	0.007	0.022	0.511	0.006	0.011
unilateral posterior bite	0.008	0.016	0.027	0.517	0.013	0.011
bilateral canine bite	0.017	0.022	0.023	0.234	0.006	0.057
unilateral posterior bite	0.021	0.027	0.042	0.193	0.010	0.062
Diademodon tetragonus						
unilateral canine bite	0.013	0.008	0.008	0.313	0.006	0.002
unilateral posterior bite	0.002	0.002	0.003	0.110	0.006	0.002
bilateral canine bite	0.013	0.008	0.008	0.257	0.007	0.001
unilateral posterior bite	0.004	0.001	0.004	0.200	0.003	0.003
Chiniquodon sanjuanensis						
unilateral canine bite	0.009	0.006	0.015	0.423	0.004	0.005
unilateral posterior bite	0.020	0.016	0.012	0.261	0.003	0.013
bilateral canine bite	0.015	0.015	0.038	0.684	0.063	0.014
unilateral posterior bite	0.023	0.024	0.035	0.631	0.058	0.013
Probainognathus jenseni						
unilateral canine bite	0.038	0.014	0.019	2.028	0.008	0.020
unilateral posterior bite	0.025	0.019	0.016	1.420	0.014	0.015
bilateral canine bite	0.031	0.009	0.016	1.917	0.009	0.029
unilateral posterior bite	0.003	0.010	0.009	1.111	0.007	0.020
Morganucodon oehleri						
unilateral canine bite	0.017	0.006	0.009	0.973	0.068	0.013
unilateral posterior bite	0.020	0.160	0.073	0.702	0.007	0.111
bilateral canine bite	0.018	0.043	0.137	0.868	0.003	0.009
unilateral posterior bite	0.016	0.037	0.012	0.813	0.007	0.016
Hadrocodium wui						
unilateral canine bite	0.019	0.016	0.038	1.900	0.013	0.022
unilateral posterior bite	0.057	0.016	0.052	3.227	0.029	0.054
bilateral canine bite	0.028	0.172	0.035	3.148	0.011	0.031
unilateral posterior bite	0.010	0.167	0.007	2.239	0.004	0.018
Monodelphis domestica						
unilateral canine bite	0.030	0.012	0.018	0.620	0.012	0.006
unilateral posterior bite	0.006	0.007	0.005	0.351	0.004	0.003
bilateral canine bite	0.027	0.148	0.027	0.594	0.011	0.003
unilateral posterior bite	0.007	0.148	0.020	0.361	0.006	0.004
Dasyurus hallucatus						
unilateral canine bite	0.028	0.014	0.014	0.591	0.011	0.008
unilateral posterior bite	0.006	0.003	0.005	0.335	0.006	0.005
bilateral canine bite	0.028	0.010	0.019	0.590	0.011	0.011
unilateral posterior bite	0.004	0.004	0.009	0.246	0.005	0.007
Petropseudes dahli						
unilateral canine bite	0.018	0.003	0.006	0.520	0.005	0.005
unilateral posterior bite	0.003	0.002	0.001	0.242	0.002	0.003
bilateral canine bite	0.021	0.006	0.003	0.668	0.007	0.003
unilateral posterior bite	0.012	0.003	0.003	0.448	0.001	0.003

Supplementary table 2. Euclidean distances calculated from PCs1-3 for all tested biomechanical scenarios relative to the undeformed models as used in figure 5 of the main text.

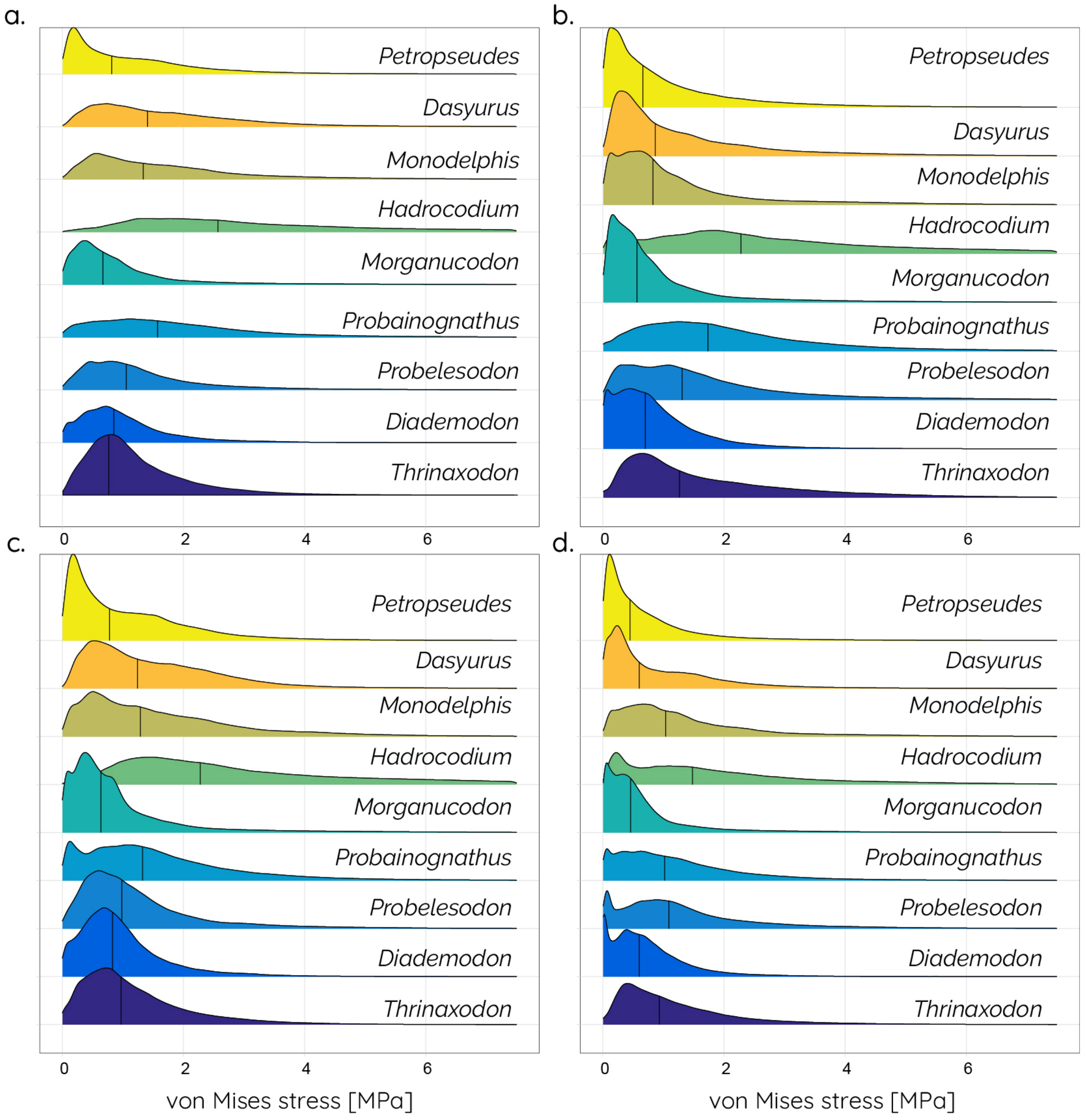

Supplementary figure 1. Ridgeline plots showing the distribution of von Mises stress in the combined skull structure (not differentiated into functional regions). (a) unilateral bite at canine, (b) unilateral bite at posterior tooth, (c) bilateral bite at canine, (d) bilateral bite at posterior tooth.

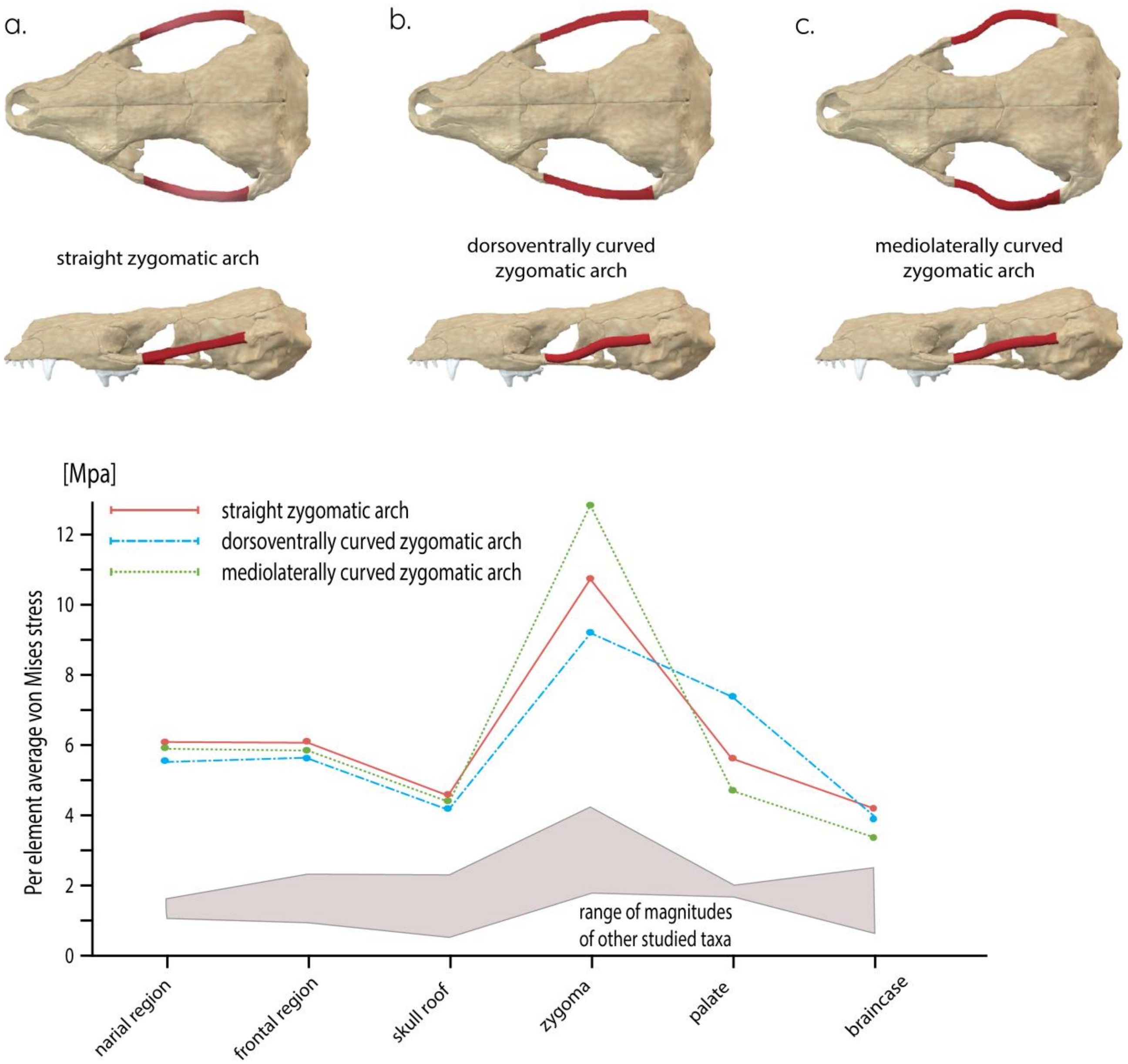

Supplementary figure 2. Comparison of functional effects of different zygomatic arch reconstructions in *Hadrocodium wui*: (a) straight zygomatic arch (most parsimonious reconstruction used in this study), (b) dorsoventrally curved zygomatic arch, (c) mediolaterally curved zygomatic arch. Difference in functional behavior shown for per element average values for each reconstruction shown.

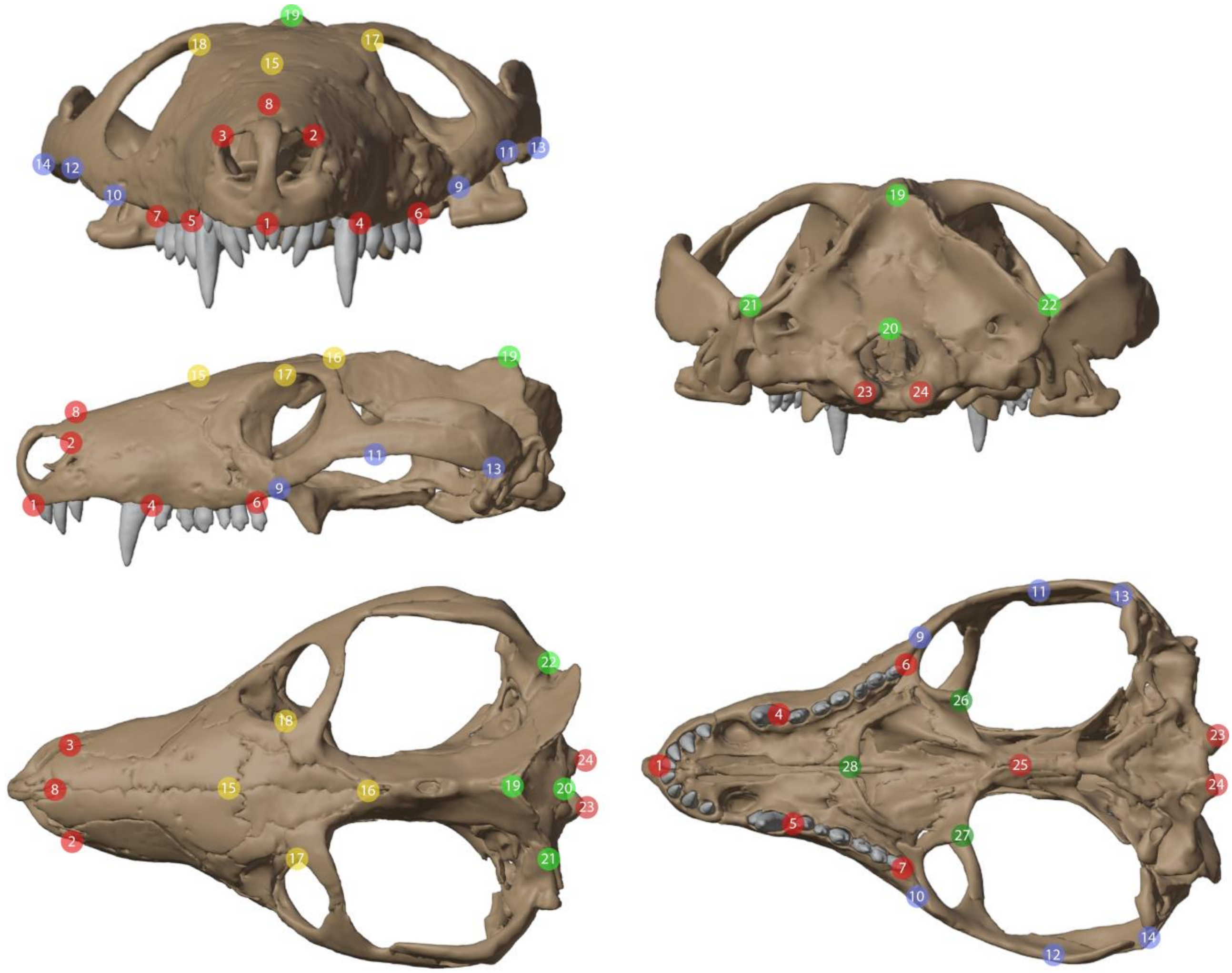

Supplementary figure 3. Landmark positions for the quantification of deformation of the different biomechanical models. Exemplified by the skull of *Thrinaxodon liorhinus* as used in figure 5 of the main text.

References cited in the supplementary information

Abdala, F., Giannini, N. Chiniquodontid cynodonts: systematic and morphometric considerations. *Palaeontology* **45**, 1151-1170 (2002).

Bonaparte, J.F., Crompton, A.W. A juvenile probainognathid cynodont skull from the Ischigualasto Formation and the origin of mammals. *Revista del Museo Argentino de Ciencias Naturales Bernardino Rivadavia* **5**, 931–936 (1994).

Kemp, T. S. *The Origin and Evolution of Mammals* (Oxford Univ. Press, Oxford, 2005).

Kermack, K. A., Mussett, F., Rigney. The skull of *Morganucodon*. *Zoological Journal of the Linnean Society* **71**, 1-158 (1981).

Lautenschlager, S. Reconstructing the past – methods and techniques for the digital restoration of fossils. *Royal Society Open Science.* **3,** 160342 (2016).

Luo, Z.-X., Crompton, A. W., Sun, A.-L. A new mammaliaform from the Early Jurassic and evolution of mammalian characteristics. *Science* **292**, 1535-1540 (2001).

Luo, Z.-X., Schultz, J. A., Ekdale, E. G. Evolution of the middle and inner ears of mammaliaforms: the approach to mammals. In (*Evolution of the Vertebrate Ear*, Springer) pp. 139-174 (2016).

Luo, Z.-X., Meng, Q. J., Grossnickle, D. M., Liu, D., Neander, A. I., Zhang, Y. G., Ji, Q. New evidence for mammaliaform ear evolution and feeding adaptation in a Jurassic ecosystem. *Nature*, **548**, 326-330 (2017).
